# Ubiquitin ligase switch in plant photomorphogenesis: A hypothesis

**DOI:** 10.1016/j.jtbi.2010.11.021

**Published:** 2011-02-07

**Authors:** Alexandra Pokhilko, Jason A. Ramos, Hans Holtan, Don R. Maszle, Rajnish Khanna, Andrew J. Millar

**Affiliations:** a School of Biological Sciences, University of Edinburgh, Mayfield Road, Edinburgh EH9 3JH, United Kingdom; b Mendel Biotechnology, Inc., 3935 Point Eden Way, Hayward, CA 94545-3720, USA; c Centre for Systems Biology at Edinburgh, C.H. Waddington Building, Kings Buildings, Mayfield Road, Edinburgh EH9 3JD, United Kingdom

**Keywords:** *Arabidopsis thaliana*, Mathematical model, Systems biology, HY5, HFR1

## Abstract

The E3 ubiquitin ligase COP1 (CONSTITUTIVE PHOTOMORPHOGENIC1) plays a key role in the repression of the plant photomorphogenic development in darkness. In the presence of light, COP1 is inactivated by a mechanism which is not completely understood. This leads to accumulation of COP1’s target transcription factors, which initiates photomorphogenesis, resulting in dramatic changes of the seedling’s physiology.

Here we use a mathematical model to explore the possible mechanism of COP1 modulation upon dark/light transition in *Arabidopsis thaliana* based upon data for two COP1 target proteins: HY5 and HFR1, which play critical roles in photomorphogenesis. The main reactions in our model are the inactivation of COP1 by a proposed photoreceptor-related inhibitor I and interactions between COP1 and a CUL4 (CULLIN4)-based ligase. For building and verification of the model, we used the available published and our new data on the kinetics of HY5 and HFR1 together with the data on COP1 abundance. HY5 has been shown to accumulate at a slower rate than HFR1. To describe the observed differences in the timecourses of the “slow” target HY5 and the “fast” target HFR1, we hypothesize a switch between the activities of COP1 and CUL4 ligases upon dark/light transition, with COP1 being active mostly in darkness and CUL4 in light. The model predicts a bi-phasic kinetics of COP1 activity upon the exposure of plants to light, with its restoration after the initial decline and the following slow depletion of the total COP1 content. CUL4 activity is predicted to increase in the presence of light. We propose that the ubiquitin ligase switch is important for the complex regulation of multiple transcription factors during plants development. In addition, this provides a new mechanism for sensing the duration of light period, which is important for seasonal changes in plant development.

## 1 Introduction

Plants undergo massive changes in the transcriptional profiles of ∼20% of their entire genome upon first exposure to light, when their developmental program switches from skotomorphogenesis in darkness to photomorphogenesis in light (Jiao et al., 2005). This causes drastic changes in plant physiology, which includes shortening of hypocotyl (embryonic stem), concomitant opening and expansion of cotyledon (embryonic leaves) and differentiation of chloroplasts (Jiao et al., 2007; Khanna et al., 2006). These changes are driven by the massive accumulation of light-responsive transcription factors, such as HY5, HFR1 and others, which were shown to be the key positive regulators of photomorphogenesis (Jiao et al., 2007; Osterlund et al., 2000; Zhang et al., 2008). The detailed mechanisms of the regulation of these transcription factors by light are largely unknown.

COP1 is a ubiquitin E3 ligase, which was shown to play a key role in the negative regulation of photomorphogenesis in darkness (Jiao et al., 2005; Ma et al., 2002; Yi and Deng, 2005). COP1 is abundant in darkness and involved in degradation of light-inducible transcription factors, such as HY5, HFR1, LAF1, BIT1 and others (Duek et al., 2004; Hong et al., 2008; Osterlund et al., 2000; Saijo et al., 2003; Seo et al., 2003). The mechanism of the regulation of COP1 activity and abundance during dark/light transition is largely unknown (Yi and Deng, 2005). It was shown that the total COP1 content in the nucleus of plant cells decreases after the transition to light, but this depletion is very slow, and takes about 24 h (von Arnim et al., 1997). To explain the observed fast accumulation of some COP1 targets, such as HFR1 or LAF1, by 2 h of illumination (Duek et al., 2004; Jang et al., 2007), the quick inactivation of COP1 by light was proposed (Yi and Deng, 2005). It has been suggested that the large multi-protein COP1 complex changes its conformation after light-induced modifications of COP1-bound components, such as the photoreceptor CRY1, which is phosphorylated after the exposure of plants to light (Li and Yang, 2007; Wang et al., 2001; Yang et al., 2001; Yi and Deng, 2005). The fast change in COP1 conformation after the dark-to-light transition could cause the observed light-induced re-distribution of the components of the CSN (COP9 signalosome) and CDD (COP10–DDB1–DET1) complexes, which interact with the COP1 complex (Chamovitz et al., 1996; Chen et al., 2010; Saijo et al., 2003; Suzuki et al., 2002; Wei et al., 2008, 1994; Yanagawa et al., 2004). This includes the dissociation of the CDD elements COP10 and DDB1 from COP1 (Chen et al., 2010; Suzuki et al., 2002; Yanagawa et al., 2004). DDB1, on the other hand, is necessary for the activation of another E3, CULLIN4-based ligase complex (Chen et al., 2010, 2006).

The CUL4 ligase complex (referred to CUL4 in this paper for brevity) is a member of the cullin RING ligases (CRLs). It contains a cullin protein, substrate recognition proteins and RING protein, which binds to an E2 ubiquitin-conjugating enzyme (Bosu and Kipreos, 2008). CUL4 is activated by neddylation and inactivated through de-neddylation by the CSN (Lyapina et al., 2001; Schwechheimer et al., 2001; Wei et al., 2008). The observed fast exclusion of DDB1 from CSN complexes after the transition to light and binding of DDB1 to CUL4 suggest that the CSN dissociates from the CUL4–DDB1 complex after the transition to light (Chamovitz et al., 1996; Wei et al., 1994, Chen et al., 2010), which should result in CUL4 activation. Thus, the available data suggest that the transition to light might lead to CUL4 activation in parallel to COP1 inactivation. CUL4 was shown to cooperate with COP1 in targeting the degradation of light-inducible transcription factors, such as HY5, but the mechanism of COP1 and CUL4 interactions is largely unknown (Chen et al., 2010, 2006). Here we used mathematical modeling as a useful tool for the study of the possible kinetics of COP1 and CUL4 activities upon dark/light transition.

Our model is based on a hypothesis that COP1 and CUL4 ligases switch their activities upon dark/light transition through mutual suppression mechanisms, so that COP1 is active mostly in darkness, but CUL4 in light. The model describes the inactivation of COP1 by light through a photoreceptor-related inhibitor I, which results in the activation and accumulation of CUL4 and slow depletion of COP1. We analyze the possible impact of the COP1/CUL4 switch on the accumulation of light-inducible transcription factors, such as HY5 and HFR1. The model was verified using quantitative published data on HFR1 protein (Duek et al., 2004), qualitative data of COP1 abundance (von Arnim and Deng, 1994; von Arnim et al., 1997) and our new quantitative data on *HY*5 mRNA and protein kinetics during dark/light transition. The observed differences in HY5 and HFR1 protein kinetics are explained by the mutual inhibition of COP1 and CUL4 and their differential efficiencies towards degradation of the target proteins. The proposed new mechanism of the ligase switch provides a basis for the diverse kinetics of light-regulated transcription factors during seedling development and plant growth under different light conditions.

## 2 The experimental verification of kinetics of *HY5* mRNA and protein upon dark-to-light transition

**Fig. 1:**
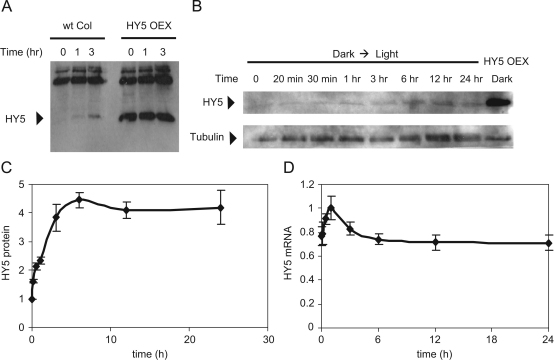
Timecourse of HY5 protein and mRNA upon dark-to-light transition. Seedlings were grown for 4 days in darkness and transferred to constant light at time 0. A: A western blot of protein extracts from wt and HY5-overexpressor line, probed with anti-HY5. B: A representative anti-HY5 western blot from wt seedlings, which were grown in darkness for 4 days and then transferred to light at time 0. Protein extracts were done at indicated time points. Tubulin protein was used as a loading control. The experiments were repeated three times with similar results. C: Quantification of the western blot, shown in B. Prior to quantification, the quantitative linear range of detection was determined by a series of dilutions on Western blots as described previously (Khanna et al., 2007). D: *HY*5 expression was analyzed by real-time PCR after dark-to light transition (see Experimental Methods in Appendix).

HY5, a bZIP transcription factor, plays a central role in plant photomorphogenesis. HY5 regulates transcription of multiple genes through binding to G-box elements in their promoters (Jiao et al., 2007). The abundance of HY5 directly correlates with the extent of photomorphogenic development (Osterlund et al., 2000). To quantitatively measure the kinetics of HY5 protein accumulation after the dark-to-light transition in Arabidopsis seedlings, we produced rabbit polyclonal antibodies against a peptide corresponding to 54–68 amino acids of the HY5 protein. Western blot analysis demonstrates the HY5 antibodies were able to detect HY5 protein of the expected size in the total protein extracts of both wt and HY5-OX seedlings upon dark-to-light transition (Fig. 1A), and the indicated band was not detectable in a *hy*5-mutant (data not shown). Next we measured the timecourse of HY5 protein accumulation after dark-to-light transition. Representative western blot analysis and the quantification of the HY5 accumulation kinetics are shown in Fig. 1B, C. Real-time PCR demonstrated the fast transient increase of *HY*5 mRNA level within 2 h, with a peak at 1 h after the transition of dark-grown seedlings to light (Fig. 1D).

## 3 Simple mathematical model—Scheme1. Simulation of HY5 and HFR1 protein kinetics upon dark-to-light transition

The kinetics of COP1 and CUL4 activities upon dark/light transition are largely unknown. It is likely that bulk levels of COP1 and CUL4 proteins do not reflect the active sub-population, so the available data on total COP1 or CUL4 may be uninformative. A suitable mathematical model, however, can use data on the abundance of target transcription factors to provide useful information about the relevant ubiquitin ligase activities. Among the multiple targets of COP1, HY5 is known to play a critical role downstream of various photoreceptors during photomorphogenesis and HFR1 is involved in phytochrome A mediated signal transduction (Fankhauser and Chory, 2000; Jenkins, 2009; Jiao et al., 2007; Vandenbussche et al., 2007; Zhang et al., 2008). In addition to their importance for plant physiology, HY5 and HFR1 protein kinetics is relatively well studied (this paper, Duek et al., 2004; Osterlund et al., 2000), which makes them good candidates for our modeling studies. The following kinetic data on HY5, HFR1 and COP1 abundance were used to build and verify the model:

(1) Quantitative kinetics of HFR1 protein upon dark-to-light transition (Duek et al., 2004).
(2) Quantitative kinetics of HFR1 protein upon light-to-dark transition (Duek et al., 2004).
(3) Quantitative kinetics of HY5 protein upon dark-to-light transition (Fig. 1C).
(4) Quantitative kinetics of *HY*5 mRNA upon dark-to-light transition (Fig. 1D).
(5) Qualitative data on HY5 protein kinetics upon light-to-dark transition (Osterlund et al., 2000).
(6) Qualitative descriptions of the timecourse of COP1 accumulation upon dark-to-light transition based on fluorescent imaging (von Arnim and Deng, 1994; von Arnim et al., 1997).
(7) Qualitative data on COP1 accumulation upon light-to-dark transition (von Arnim and Deng, 1994; von Arnim et al., 1997).

**Fig. 2:**
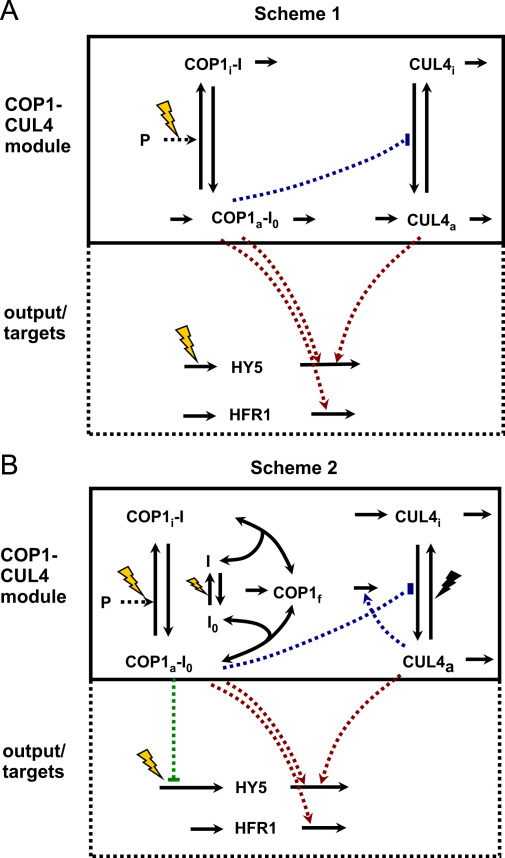
Schematic description of the mathematical models of the ligase switch. Light inputs are shown by yellow flashes, which stimulate *HY*5 expression and inactivation of COP1 activity through the activation of inhibitor I_0_ in complex with protein P. Activated inhibitor is denoted as I. Arrows without starting or terminal substances correspond to production/translation or degradation of the corresponding proteins. The targeted degradation of HY5 and HFR1 proteins by the active ligases is shown by red dotted lines. A: Simple model (Scheme 1) with only one-sided negative regulation of CUL4 by active COP1 (blue dotted connection). B: Full model (Scheme 2) with two-sided mutual negative regulation of COP1 and CUL4 (blue lines). The dissociation of the COP1-inhibitor complexes and targeted degradation of the free COP1 (COP1_f_) by CUL4 are included. Negative regulation of *HY*5 expression by COP1 is shown (green line). The accelerated inactivation of CUL4 ligase in darkness is shown by the black flash. The detailed scheme of the full model in SBGN format is given in Fig. 7 of the Appendix.

The experimental data demonstrated the essential differences between HY5 and HFR1 protein kinetics after the transition of dark-grown seedlings to light with: (1) faster accumulation of HFR1 than HY5, (2) sharp fall of HFR1 immediately after its rise (Duek et al., 2004), in contrast to the slower, saturated HY5 kinetics (Fig. 1C). Our preliminary simulations of a model with only one ligase (COP1) resulted in a failure to describe the observed differences in HY5 and HFR1 kinetics (not shown). Then we introduced the CUL4 ligase, which was shown to participate in HY5 degradation together with COP1 (Chen et al., 2006). Light is known to inactivate COP1 through the activation of COP1-bound photoreceptors, such as CRY1 in blue light and phytochromes in red light (Li and Yang, 2007; Wang et al., 2001; Yang et al., 2001; Yi and Deng, 2005). In our model we used the generic name I_0_ for these photoreceptor-related inhibitors of COP1. The activated inhibitor I caused inactivation of the bound COP1 through conformational changes in COP1 molecule (Fig. 2A; Yi and Deng, 2005). The transience of the activation of inhibitor I_0_ by light was modeled analogous to Locke et al. (2006) by introducing light-regulated protein P, which was necessary for the activation of I_0_ by light. The protein P represented a photoreceptor-bound component, such as a PIF protein, which is produced in darkness and degraded in light (Monte et al., 2007). Next we hypothesized that active COP1 suppresses CUL4 activity (Fig. 2A). A more detailed mechanism of this suppression could be realized through the formation of multi-protein complexes of the active COP1, COP10/DDB1 and CSN (Chen et al., 2006; Suzuki et al., 2002) and the inhibition of the CUL-based ligase by CSN (Wei et al., 2008). Inactivation of COP1 by light could cause re-distribution of CSN, COP10/DDB1 and COP1 complexes (Chamovitz et al., 1996; Saijo et al., 2003; Suzuki et al., 2002; Wei et al., 2008, 1994; Yanagawa et al., 2004) and the release of CUL4 from suppression. We simplified the mechanism by assuming that only CUL4 and COP1 activities are regulated upon dark/light transitions.

The model was built in two steps. At the first step we considered a simple scheme of reactions with only one-sided negative regulation of CUL4 activity by active COP1 (Scheme 1 of Fig. 2A; model equations are presented in Appendix). We tested the model to determine whether a scheme of this type can account for the data. The model included two modules: (1) COP1-CUL4 interactions and (2) the output module of the target proteins HY5 and HFR1 (Fig. 2A). The rate of HFR1 translation was assumed to be constant based on available data on *HFR*1 mRNA abundance (Duek and Fankhauser, 2003). The level of *HY*5 mRNA, however, is known to change quickly upon dark/light transition (Fig. 1D; Osterlund et al., 2000), so we included a separate equation for *HY*5 mRNA to describe the observed light-induced changes in *HY*5 expression (see Appendix).

**Fig. 3:**
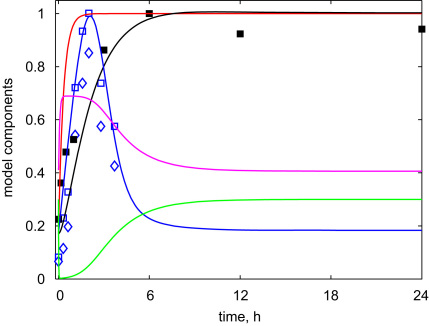
The simulated kinetics of the simple model (Scheme 1) upon dark-to-light transition. The activities of COP1 and CUL4 ligases are shown by green and magenta lines, respectively. The kinetics of HFR1 and HY5 proteins and *HY*5 mRNA are shown by blue, black and red lines, respectively. Experimental points for HFR1 protein (blue) are taken from (Duek 04) and for HY5 protein (black)—from this paper (Fig. 1C). The simulation was run starting from initial conditions, which correspond to the steady state of the system in darkness: *c*_*COP*1*i*_=0.2; *c*_*COP*1*a*_=0.3; *c*_*P*_=1; *c*_*CULi*_=0.594; *c*_*CULa*_=0.406; $c_{HY5}^{m}=0.167$; *c*_*HY*5_=0.167; *c*_*HFR*1_=0.183.

After the fitting of the model parameters (shown in Table A1 of the Appendix), Scheme 1 was able to simulate correctly the experimental kinetics of HFR1 and HY5 proteins upon the dark-to-light transition (Fig. 3). The model explained the experimentally observed quick rise and the following fall of HFR1 protein level through the opposite fall and rise of COP1 activity (Fig. 3). However, the simulated HY5 protein showed less dependence on COP1, demonstrating slower saturated kinetics, which was related to the additional impact of CUL4 on HY5 kinetics during the fast initial rise of CUL4 activity at 0.5–2 h after lights-on (Fig. 3). The model explained the difference between HFR1 and HY5 protein kinetics through a higher rate constant of HFR1 degradation by COP1 compared to HY5.

Although Scheme 1 matched the kinetics of COP1 and CUL4 targets upon dark-to-light transition, it failed to describe several other experimental observations. First, the steady-state level of the total COP1 in the model was the same in light and darkness, which did not reflect the experimental observation of the lower COP1 in light-grown seedlings compared to the dark-grown seedlings (von Arnim and Deng, 1994; von Arnim et al., 1997). And second, Scheme 1 did not include the observed negative regulation of *HY*5 expression by COP1 (Oyama et al., 1997) and could not describe the observed noticeable fall in *HY*5 mRNA level immediately after its rise (Fig.1D). At the next step we extended the reactions of Scheme 1 to account for these experimental observations.

## 4 Evolution of the mathematical model. Full model—Scheme 2. COP1/CUL4 kinetics upon dark-to-light transition

Inactivation of COP1 by light was the source of light input into the COP1–CUL4 module in Scheme 1, which provided a transient response of the system to the change in light conditions. To describe the observed differences in the steady-state levels of total COP1 in light and darkness, we included: 1—regulation of CUL4 activity by light and 2—targeted degradation of free COP1 by CUL4. Additional regulation of CUL4 activity was introduced by analogy with the sequestration of cullins by CAND1 protein, which is necessary for the cycling of cullin activity (Bosu and Kipreos, 2008). Although the details of the regulation of CUL4 activity by CAND1 and CSN in plants are not fully understood (Chen et al., 2006), the sequestration of inactive CUL4 by CAND1 after inactivation of CUL4 by CSN was found in other organisms (Bosu and Kipreos, 2008). The differences in the molecular weights of the “light” and “dark” CSN complexes in plants, re-distribution of CSN complexes with COP1 and COP10 upon light/dark transitions and strong phenotype of *csn* mutants in darkness suggested that CSN activity increased in darkness (Chamovitz et al., 1996; Suzuki et al., 2002; Wei et al., 1994). This should result in inactivation of CUL4, followed by sequestration of inactive CUL4 by CAND1. The absence of data on the changes in the structure of CUL4 complexes with CSN and CAND1 upon dark/light transitions preclude the explicit modeling of these interactions at present. However, we included the acceleration of CUL4 inactivation in darkness, which would result from interactions of this type. The assumption about the targeted degradation of the free COP1 by CUL4 was based on the observed ubiquitination of COP1 (Saijo et al., 2003; Seo et al., 2003; Yi and Deng, 2005); association of CUL4 with COP1 complexes (Chen et al., 2010, 2006) and depletion of COP1 content in the presence of high CUL4 activity in *csn* mutants (Chamovitz et al., 1996; von Arnim et al., 1997). In addition, we included the observed negative regulation of *HY*5 expression by COP1 (Oyama et al., 1997). The dissociation of COP1–inhibitor complexes was also taken into consideration. The rate of COP1 translation was assumed to be constant based on the observed absence of regulation of *COP*1 expression by light (Deng et al., 1992). Additionally, we assumed that CUL4 translation and degradation does not change during dark/light transition, based on the data on similar levels of CUL4 protein in the light and darkness (Chen et al., 2010). The full Scheme 2 of reactions is shown in Fig. 2B.

**Fig. 4:**
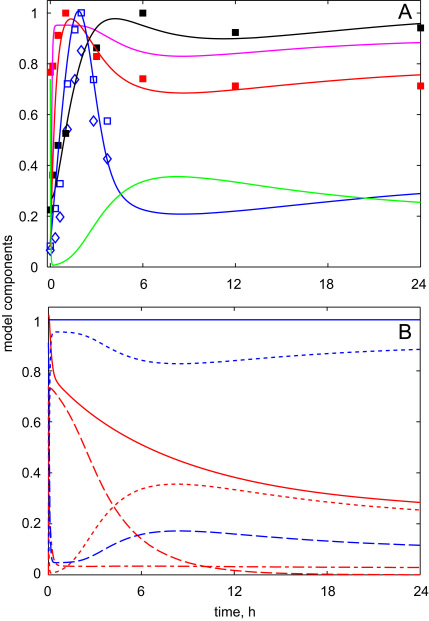
The simulated kinetics of the full model (Scheme 2) upon dark-to-light transition. A: The activities of COP1 and CUL4 ligases are shown by green and magenta lines, respectively. The kinetics of HFR1 and HY5 proteins and *HY*5 mRNA are shown by blue, black and red lines, respectively. Experimental data points—as in Fig. 3. B: The kinetics of different forms of COP1 (red) and CUL4 (blue): Active forms are shown by dotted lines, inactive forms—by dashed lines, free COP1—by dashed-dotted line, total content—by solid lines. The simulation was run starting from initial conditions, which correspond to the steady state of the system in darkness: *c*_*COP*1*i*_=0; *c*_*COP*1*a*_=0.737; *c*_*I*_=0; *c*_*P*_=1; *c*_*COP*1*f*_=0.28; *c*_*CULi*_=0.911; *c*_*CULa*_=0.089; $c_{HY5}^{m}=0.081$; *c*_*HY*5_=0.266; *c*_*HFR*1_=0.1.

After fitting the model parameters (Table A2 of the Appendix), Scheme 2 closely matched experimental data during the transition of dark-grown seedlings to light. The description of the output module was improved compared to Scheme 1 through the better simulation of the experimentally observed immediate fall of *HY*5 mRNA after its initial rise (Fig. 4A). The model explained this fall by the restoration of COP1 activity, which potentially had negative impact on *HY*5 expression. Scheme 2 also described correctly the observed kinetics of HFR1 and HY5 proteins (Fig. 4A) through the mechanism, analogous to Scheme 1 (Fig. 3), which is based upon higher activity of COP1 towards HFR1 than HY5. In contrast with Scheme 1, Scheme 2 described the experimentally observed slow fall in the total COP1 content after the transition of plants to light (von Arnim et al., 1997). Fig. 4B shows the kinetics of the different forms of COP1 and CUL4 together with their total contents. Importantly, the sharp changes in the ligase activities after lights-on were related with their re-distribution between different forms, while the total ligase content changed more slowly. The substantial decline in the simulated COP1 content after 24 h of light corresponded to the experimental observation (von Arnim and Deng, 1994; von Arnim et al., 1997).

In addition to the description of the available experimental data, the model demonstrated two new properties of the dark-to-light transition. First, the experimentally observed fall in HFR1 protein after its initial rise suggested that HFR1 was degraded again after 2–5 h in light. This fall could not be described by the much slower fluctuations in *HFR*1 mRNA expression (Duek and Fankhauser, 2003). Thus, the HFR1 data constrained the model dictating a restoration of COP1 activity after its transient inactivation by light. The resulting kinetics of COP1 activity was predicted to be bi-phasic with its restoration by 6 h after the initial sharp fall at 10 min (0.16 h) after lights-on (Fig. 4A). Second, the model predicted the increase of CUL4 activity in the presence of light, which resulted from inactivation of COP1 and decrease in CUL4 inactivation (Fig. 4B).

## 5 Simulated kinetics of light-to-dark transition. Restoration of COP1 content

**Fig. 5:**
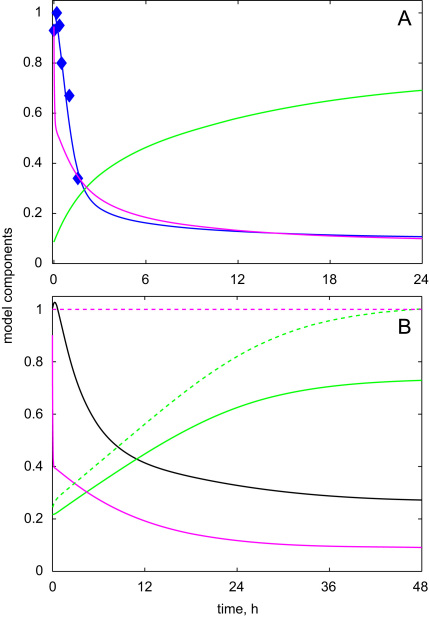
The simulated kinetics of the full model upon light-to-dark transitions. A: The activities of COP1 (green) and CUL (magenta) ligases and HFR1 protein kinetics (blue) after the transition of dark-grown plants, which were exposed to 2h of light, back to darkness. The data for HFR1 protein was taken from (Duek et al., 2004). The initial conditions, which correspond to the state of the system after 2 h of light, were *c*_*COP*1*i*_=0.549; *c*_*COP*1*a*_=0.086; *c*_*I*_=0.358; *c*_*P*_=0.018; *c*_*COP*1*f*_=0.033; *c*_*CULi*_=0.055; *c*_*CULa*_=0.945; $c_{HY5}^{m}=0.946$; *c*_*HY*5_=0.814; *c*_*HFR*1_=1. B: The kinetics of HY5 protein (black) after the transition of the light-grown plants to darkness. The activities of CUL4 and COP1 ligases are shown by magenta and green solid lines, respectively. The total CUL4 and COP1 contents are shown by dashed line. The simulation was run starting from initial conditions, which correspond to the steady state of the system in the presence of light: *c*_*COP*1*i*_=0; *c*_*COP*1*a*_=0.217; *c*_*I*_=0; *c*_*P*_=0; *c*_*COP*1*f*_=0.028; *c*_*CULi*_=0.098; *c*_*CULa*_=0.902; $c_{HY5}^{m}=0.789$; *c*_*HY*5_=1.005; *c*_*HFR*1_=0.341.

Next we simulated the system kinetics in darkness, after the transition of plants from light. Fig. 5A shows that the model described correctly the experimentally observed fast fall in HFR1 protein level in darkness after exposure of plants to 2 h of light (Duek et al., 2004). The model explained this fall by the restoration of COP1 activity in darkness (Fig. 5A). HY5 protein was depleted more slowly in darkness (Fig. 5B). This matched the available western blot data, which showed the substantial decline of HY5 protein content in light-grown plants after 15 h of darkness (Osterlund et al., 2000). Fig. 5B also demonstrates the slow restoration of COP1 activity and content, which saturated after one day of darkness, in agreement with the experiments (von Arnim and Deng, 1994). The model showed a fall in CUL4 activity in darkness, while CUL4 content did not change (Fig. 5B).

The slow decrease of total COP1 content in the presence of light and its increase in darkness in our model is related with the higher rate of COP1 degradation by CUL4 in light compared to darkness. Further experiments on the kinetics of CUL4 activity under various light conditions are necessary to test this prediction of the model. In addition to the regulation of COP1 by its differential degradation, light affects the re-distribution of COP1 between nucleus and cytoplasm through specific nuclear export/import (Schwechheimer et al., 2001; von Arnim et al., 1997). Quantitative measurements of the nuclear versus cytoplasmic COP1 contents are necessary for further inclusion of this additional mechanism of COP1 regulation into the model.

## 6 “Fast” and “slow” COP1 substrates. Simulation of the photoperiodic regulation of COP1–CUL4 ligase switch. Dawn/dusk sensing by COP1 and CUL4 targets

**Fig. 6:**
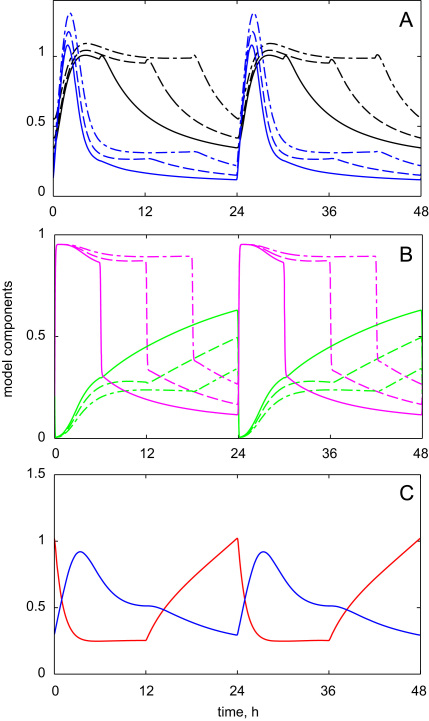
Simulated kinetics of the model components under various photoperiods. The simulations were initially run for 4 days under each photoperiod to entrain the system, so that only 5th and 6th days are shown. A, B: Solid, dashed and dashed-dotted lines correspond to 6 L:18 D, 12 L:12 D and 18 L:6 D light–dark cycles, respectively. Blue, black, green and magenta colors show the kinetics of HFR1, HY5 proteins and the activity of COP1 and CUL4 respectively. C: Simulated kinetics of the hypothetical COP1 (blue) and CUL4 (red) substrates under 12L:12D. HFR1 equation was used for COP1 substrate with the following parameter values: *p*_5_=0.28 h^−1^; *h*_7_=2 h^−1^. HY5 protein equation was used for CUL4 substrate with constant expression level *p*_4_ and the following parameter values: *p*_4_=0.22 h^−1^; *h*_4_=1 h^−1^; *h*_5_=0. The rest of the parameters are shown in Table 2 of the Appendix.

HFR1 and HY5 proteins represented two classes of COP1 targets with fast and slow kinetics, respectively. The observed accumulation of HY5 after transition of plants to light and its depletion in darkness was much slower than for HFR1 (Figs. 4 and 5). The model explained these experimental observations by the higher efficiency of COP1-mediated degradation of the “fast” substrates, such as HFR1. The differences in the kinetics of the fast and slow COP1 targets were further demonstrated by simulations of diurnal conditions with various photoperiods. Fig. 6A shows the fast transient accumulation of HFR1 protein in the morning under all photoperiods, which resulted from the transient fall and then restoration of COP1 activity (Fig. 6B). HY5 protein had slower kinetics and stayed at high level during the whole light period (Fig. 6A), when *HY*5 expression is high. Thus the model predicted a higher level of HY5 protein under long summer days (18 h of light) compared to the short winter days (6 h of light) (Fig. 6A), which would result in the prolonged stimulation of downstream processes, such as anthocyanin biosynthesis (Ang et al., 1998). We also demonstrated the opposite regulation of COP1 and CUL4 activities by light, which resulted in decrease of COP1 activity and increase of CUL4 activity in light and the opposite trends in darkness (Fig. 6B). The model predicted an increase of the maximal level of COP1 activity in short days compared to long days (Fig. 6B).

The results above focus on the dynamic profiles of COP1 target proteins after light/dark transitions. The ligase switch also allows the plants to sense changes in light conditions through the accumulation of COP1 substrates in the day time and CUL4 substrates at night. Fig. 6C demonstrated this idea with hypothetical COP1 and CUL4 substrates. For example, COP1 was found to be involved in the regulation of important flowering regulators CO and GI (Jang et al., 2008; Yu et al., 2008). Interestingly COP1 degrades both CO and GI proteins only at night, while some other unknown ligase degrades them in the day time (Jang et al., 2008; Yu et al., 2008). Further experiments are necessary to investigate the possible effect of CUL4 on the degradation of the flowering components. The functional relationship between various ligases and the circadian clock has started to emerge recently through discoveries connecting COP1 and some of the important circadian elements GI and ELF3 (Yu et al., 2008), and between F-box proteins ZTL and circadian proteins TOC1 and PRR5 (Kiba et al., 2007; Kim et al., 2007).

The mechanism of interaction between COP1 and CUL4 is still unknown (Chen et al., 2010). The differences in molecular weights of COP1 (∼700 kD; Saijo et al., 2003; Yanagawa et al., 2004) and CUL4 (∼400 kD, Chen et al., 2010) complexes suggests a difference in composition of COP1 and CUL4 complexes. Indeed, recent studies showed that no COP1–CUL4 supercomplex was found, which was explained through the existence of distinct COP1 and CUL4 complexes (Chen et al., 2010). However, it remained unclear, why the CUL4–DDB1 complex was found to be directly associated with COP1 *in vivo* (Chen et al., 2010, 2006). In our model we hypothesized that dissociation of the COP1 complex can result in binding of free COP1 molecules to active CUL4 and result in the degradation of COP1. Experimental verification of the hypothesized degradation of COP1 by CUL4 may be complicated by the auto-ubiquitination of COP1. Future measurements of the CUL4 ligase activity towards inactive mutated COP1 would resolve this problem.

Our minimal model of the regulation of COP1/CUL4 activities by light is sufficient to describe the existing data on HY5 and HFR1 kinetics. The model incorporates the regulation of COP1 complexes with inhibitor I by light, which is crucial for the kinetics of the system upon dark/light transitions. However, the whole system of COP1/CUL4 regulation in plants includes more elements, which are required for the fine-tuning of COP1 and CUL4 activity in various plant organs and under different qualities of light. The mechanisms of the interaction between the multiple elements of the system during the dark/light transition are largely unknown. In particular, SPA proteins provide additional levels of regulation of COP1 activity towards different targets (Saijo et al., 2003; Seo et al., 2003; Zhu et al., 2008) and four members of the SPA protein family have multiple effects on COP1 activity under various light qualities (Fittinghoff et al., 2006). For example, SPA1 modulates COP1 activity towards degradation of HFR1, which is important under far red and blue light conditions (Fankhauser and Chory, 2000; Yang et al., 2005). SPA genes are quickly expressed after the dark-to-light transition in a photoreceptor-dependent manner (Fittinghoff et al., 2006) and heteromeric complexes of SPA proteins with COP1 have diverse effects of COP1 protein abundance (Zhu et al., 2008). The mechanisms of opposite effects of different SPA proteins on COP1 are not known. Additionally, it was shown that there is a redundancy in SPA functions in plants, with spa triple and quadruple showing the strongest phenotypes (Fittinghoff et al., 2006). More recent studies demonstrated that the changes in SPA protein concentrations are relatively slow compared to the fast changes in the kinetics of COP1 targets (Zhu et al., 2008). This suggests that the fast changes in COP1/CUL4 system upon dark/light transitions are mainly determined by some other components of the system, such as CSN, CDD and CAND1. Moreover, COP1 was shown still to aggregate into large complexes (∼700 kD) in the absence of all four SPA proteins (Zhu et al., 2008). The absence of clear mechanisms of the diverse SPA functions and high complexity of multiple SPA–COP1 interactions precluded the inclusion of SPA proteins in our minimal model of COP1 regulation. In the future, data on regulation of the composition of COP1 and CUL4 complexes with CSN, CDD and CAND1 by light should allow us to include additional components into the next model.

In conclusion, this is the first mathematical model of the regulation of COP1 and CUL4 ligase activities by an input signal, in this case light. The model explains the dynamics of the accumulation of target proteins through the interaction of the input signal with a molecular mutual-inhibition mechanism, as discussed in (Van Cauter et al., 1976). In the model COP1 is regulated by light through two parallel mechanisms: COP1 is quickly inactivated after lights-on through the modification of inhibitor I, and COP1 abundance slowly decreases in the presence of light through up-regulation of CUL4. The fall in COP1 activity after lights-on allows accumulation of the “fast” COP1 targets, such as HFR1 (Fig. 4A). We also predicted some restoration of COP1 activity after its initial fall, which is necessary for the quick down-regulation of the “fast” COP1 targets (Figs. 4A, 6A). The parallel activation of CUL4 activity in presence of light provided the additional mechanism required for the regulation of “slow” COP1 substrates, such as HY5, which stayed high during the whole period in light (Figs. 4A, 6A). Interestingly, both COP1 and CULLIN4 ligases are broadly present in most organisms, so the proposed mechanism of the ligase switch may be applicable to other biological processes as well. Finally, the model suggests a new mechanism of light perception by the ligase switch in plants.

## Figures and Tables

**Table A1:** The optimal parameter set for the Scheme 1.

Parameter	*k*_0_	*k*_1_	*k*_6_	*k*_7_	*k*_8_	*k*_9_	*p*_1_
Value (h^−1^)	75	0.3	3	10	3	0.5	0.6

Parameter	*p*_2_	*p*_3_	*p*_4_	*p*_5_	*h*_1_	*h*_2_	*h*_3_
Value (h^−1^)	0.1	1.5	0.42	0.55	2	0.2	3

Parameter	*h*_4_	*h*_5_	*h*_7_	*h*_9_	*g*_1_	*a*	
Value	0.44 h^−1^	0.8 h^−1^	10 h^−1^	1.5 h^−1^	0.2	2	

**Table A2:** The optimal parameter set for the Scheme 2.

Parameter	*k*_0_	*k*_1_	*k*_2_	*k*_3_	*k*_4_	*k*_5_	*k*_7_	*k*_8_
Value (h^−1^)	47	0.3	0.1	1	0.1	1	20	3.1

Parameter	*k*_9_	*h*_1_	*h*_2_	*h*_3_	*h*_4_	*h*_5_	*h*_7_	*h*_8_
Value (h^−1^)	0.5	2	8	3	0.5	0.2	10	13

Parameter	*h*_9_	*p*_1_	*p*_2_	*p*_3_	*p*_4_			
Value (h^−1^)	1	0.3	0.2	1	0.63			

Parameter	*p*_5_	*g*_1_	*g*_2_	*g*_3_	*a*			
Value	0.74 h^−1^	0.2	0.7	0.05	2			

**Fig. 7:**
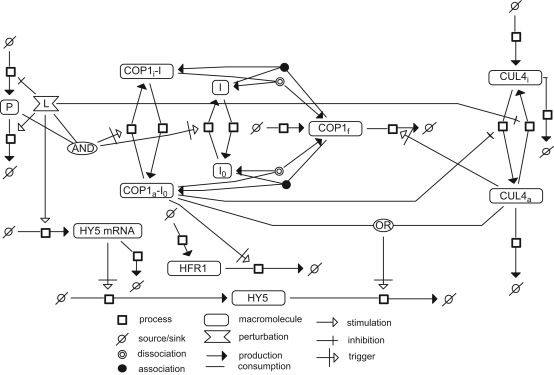
The reaction scheme of the full model in SBGN format. The scheme was draw using Edinburgh Pathway Editor (EPE), which is freely available from http://sourceforge.net.

**Fig. 8:**
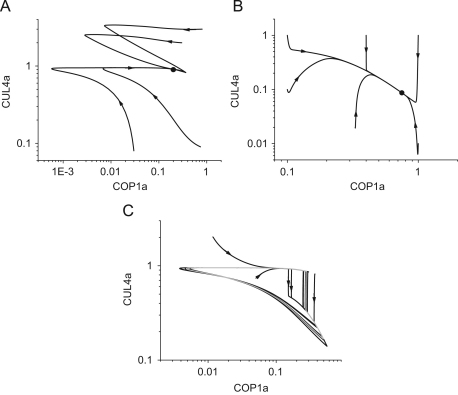
The phase diagrams of the full system under various light conditions. The panels correspond to the constant light (A), constant darkness (B) and diurnal 12 L:12 D light/dark cycles (C). The direction of the trajectories are shown by arrows, black points correspond to steady states and limit cycle is shown by gray line. The simulations were done in MATLAB.

**Fig. 9:**
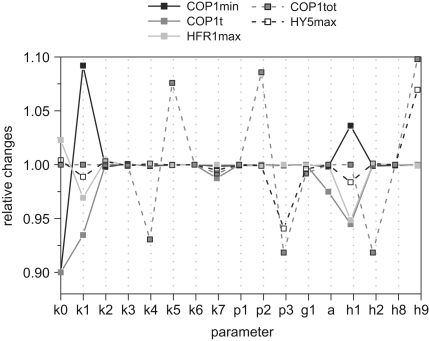
Relative changes in the kinetics of the model after dark-to-light transition for 10% increase of each parameter of the COP1/CUL4 module. The following kinetic characteristics are shown: The value of the first sharp fall in COP1 activity (COP1min) and its time (COP1t); values of the maximum of HFR1 and HY5 proteins (HFR1max and HY5max); steady state value of the total COP1 content (COP1tot).

**Fig. 10:**
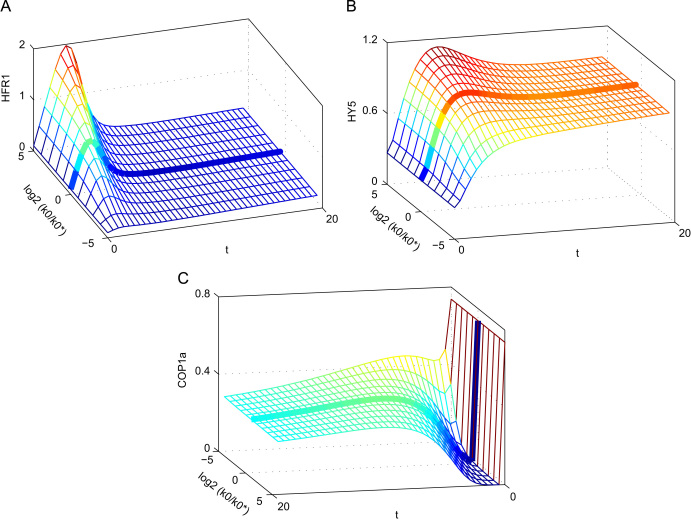
Dynamics of the model after dark-to light transition (at time 0) upon variation of the rate constant of COP1 inactivation by light (*k*_0_). Parameter *k*_0_ was increased/decreased by order of 2 from its optimal value (*k*_0_⁎, marked by thick lines). A, B, C panels show the kinetics of HFR1, HY5 proteins and COP1 activity, respectively.

## Appendix A

### A.1 Experimental Methods

#### A.1.1 Plant material and growth conditions

*Arabidopsis thaliana* ecotype Columbia (Col) seedlings were grown on plates containing growth medium. Seedlings were grown in the dark for four days and then transferred to continuous white light. Tissue samples were collected at various timepoints in the dark and in light as indicated. *HY*5 transcript levels were determined by qRT-PCR using total RNA as template, and HY5 protein levels were quantified using Western blots. All experiments were performed three times.

#### A.1.2 Quantitative immunoblot analysis

HY5 protein levels were detected and quantified from total protein extracts prepared from dark-grown or light-treated seedlings in denaturing extraction buffer (100 mMTris-HCl, pH 7.8, 4 M urea, 5% SDS, 15% glycerol and 10 mM DTT) with protease inhibitors, according to the protocol described (Khanna et al., 2007). HY5 protein was detected with a rabbit polyclonal anti-HY5 antibody. This antibody was generated against a synthetic peptide corresponding to HY5 amino acids 54–68. The polyclonal antisera were affinity-purified using the same synthetic peptide. The specificity of the affinity-purified antisera was confirmed by detecting expected size HY5 protein in wild-type Col and HY5 overexpressing seedlings (Fig. 1A), and the absence of that band in hy5-mutant seedlings. Next, we determined a linear quantitative range for detecting HY5 protein levels on western blots with our reagents by performing a dilution series with total extracts (as described in Khanna et al., 2007). Tubulin was used as a control for loading and for the quantification of HY5 protein levels.

### A.2 Simple mathematical model

The system of the ordinary differential equations (ODE), which describes the simple model of the Scheme1 of Fig. 2A, is presented below

(1) $$dc_{COP1i}/dt=p_{2}+k_{0}L\cdot c_{P}\cdot c_{COP1a}-k_{1}c_{COP1i}-h_{2}c_{COP1i}$$

(2) $$dc_{COP1a}/dt=k_{1}c_{COP1i}-k_{0}L\cdot c_{P}\cdot c_{COP1a}-h_{2}c_{COP1a}$$

(3) $$dc_{P}/dt=p_{1}D\cdot (1-c_{P})-h_{1}c_{P}L$$

(4) $$dc_{CULi}/dt=p_{3}+k_{6}c_{CULa}-k_{7}c_{CULi}\cdot g_{1}^{a}/(g_{1}^{a}+c_{COP1a}^{a})-h_{9}c_{CULi}$$

(5) $$dc_{CULa}/dt=k_{7}c_{CULi}\cdot g_{1}^{a}/(g_{1}^{a}+c_{COP1a}^{a})-k_{6}c_{CULa}-h_{9}c_{CULa}$$

(6) $$dc_{HY5}^{m}/dt=k_{8}\cdot L+k_{9}\cdot D-h_{3}c_{HY5}^{m}$$

(7) $$dc_{HY5}/dt=p_{4}c_{HY5}^{m}-h_{4}c_{HY5}c_{CULa}-h_{5}c_{HY5}c_{COP1a}$$

(8) $$dc_{HFR1}/dt=p_{5}-h_{7}c_{HFR1}c_{COP1a}$$

where $c_{COP1i};\;c_{COP1a};\;c_{P};\;c_{CULa};\;c_{CULi};\;c_{HY5}^{m};\;c_{HY5};\;c_{HFR1}$ are amount of COP1_*i*_-I and COP1_*a*_-I_0_ protein complexes, protein P, CUL4_*a*_ and CUL4_*i*_ proteins, *HY*5 mRNA, HY5 and HFR1 proteins, respectively. All concentrations are dimensionless, providing that the total levels of COP1, CUL4, P, *HY*5 mRNA, and HY5 and HFR1 proteins are normalized to their maximal levels, reaching 1 in maximum under constant light or dark conditions. *L*=1 when light is present; *D*=1−*L*. Time unit is an hour. The parameters *p*_*j*_ are constants of translation/production of proteins and their complexes; *h*_*j*_ are constants of degradation of all substances; *k*_*j*_ are constants of protein complexes formation, dissociation, modifications and *HY*5 mRNA transcription; *g*_*j*_ are Michaelis–Menten constants and *a* is a Hill coefficient. The use of Hill function for the inactivation of CUL4 by COP1 was based on the data on the formation of active COP1 dimers, which interact with CUL4 in a large multi-protein complex together with SPA proteins (Chen et al., 2010; Zhu et al., 2008). The optimal set of parameter values, which was used in simulation shown on Fig. 3, is presented in Table A1.

### A.3 Full mathematical model

The system of the ODE, which corresponds to the Scheme 2 of the full model (Fig. 2B) is presented below

(11) $$dc_{COP1i}/dt=k_{0}L\cdot c_{P}\cdot c_{COP1a}-k_{1}c_{COP1i}-k_{2}c_{COP1i}+k_{3}c_{I}c_{COP1f}$$

(12) $$dc_{COP1a}/dt=k_{1}c_{COP1i}-k_{0}L\cdot c_{P}\cdot c_{COP1a}-k_{4}c_{COP1a}+k_{5}c_{I0}c_{COP1f}$$

(13) $$dc_{P}/dt=p_{1}D\cdot (1-c_{P})-h_{1}c_{P}L$$

(14) $$dc_{I}/dt=k_{2}c_{COP1i}-k_{3}c_{I}c_{COP1f}+k_{0}L\cdot c_{P}\cdot c_{I0}-k_{1}c_{I}$$

(15) $$dc_{COP1f}/dt=p_{2}+k_{2}c_{COP1i}+k_{4}c_{COP1a}-k_{3}c_{I}c_{COP1f}-k_{5}c_{I0}c_{COP1f}-h_{2}c_{COP1f}c_{CULa}$$

(16) $$dc_{CULi}/dt=p_{3}-k_{7}c_{CULi}\cdot g_{1}^{a}/(g_{1}^{a}+c_{COP1a}^{a})+h_{8}D\cdot c_{CULa}-h_{9}c_{CULi}$$

(17) $$dc_{CULa}/dt=k_{7}c_{CULi}\cdot g_{1}^{a}/(g_{1}^{a}+c_{COP1a}^{a})-h_{8}D\cdot c_{CULa}-h_{9}c_{CULa}$$

(18) $$dc_{HY5}^{m}/dt=(k_{8}\cdot L+k_{9}\cdot D)\cdot g_{2}/(g_{2}+c_{COP1a})-h_{3}c_{HY5}^{m}$$

(19) $$dc_{HY5}/dt=p_{4}c_{HY5}^{m}-h_{4}c_{HY5}c_{CULa}-h_{5}c_{HY5}c_{COP1a}$$

(20) $$dc_{HFR1}/dt=p_{5}-h_{7}c_{HFR1}c_{COP1a}$$

where $c_{COP1i};\;c_{COP1a};\;c_{I};\;c_{I0};\;c_{COP1f};\;c_{P};\;c_{CULa};\;c_{CULi};\;c_{HY5}^{m};\;c_{HY5};\;c_{HFR1}$ are amount of COP1_*i*_-I, COP1_*a*_-I_0_ complexes, I, I_0_, free COP1, P, CUL4_*a*_, CUL4_*i*_ proteins, *HY*5 mRNA, HY5 and HFR1 proteins. The total amounts of COP1, CUL4, P, *HY*5 mRNA, and HY5 and HFR1 proteins are normalized to its maximal level. The total amount of the inhibitor was considered to be conserved: *c*_*I*_+*c*_*I*0_+*c*_*COP*1*a*_+*c*_*COP*1*i*_=1, allow to express *c*_*I*0_ through the other inhibitor-bound components. Time unit is an hour. The definition of parameters is the same as for Scheme1.

The model presents a minimal circuit for the COP1/CUL4 ligase switch. It is based on the following assumptions, which are explained in more detail in the main text, and drawn from the following references: (1) Light transiently inactivates COP1 protein through the activation of a COP1-bound inhibitor I. I reflects the action of photoreceptors, such as CRY1, on COP1 (Li and Yang, 2007; Wang et al., 2001; Yang et al., 2001; Yi and Deng, 2005); (2) COP1 protein inhibits activation of CUL4, for example through a COP10/DDB1/CSN-related mechanism (Chamovitz et al., 1996; Chen et al., 2006; Suzuki et al., 2002; Wei et al., 1994; Yanagawa et al., 2004); (3) CUL4 is inactivated in darkness, for example through a CAND1/CSN-related mechanism (Bosu and Kipreos, 2008; Chamovitz et al., 1996; Chen et al., 2006; Wei et al., 1994); (4) COP1 is targeted for degradation by CUL4 (Chamovitz et al., 1996; Chen et al., 2006; Saijo et al., 2003); (5) HY5 expression is activated by light (Fig. 1D; Osterlund et al., 2000) and inhibited by COP1 (Oyama et al., 1997); (6) COP1, CUL4 and HFR1 RNA expression is constant over the relevant timescale (Chen et al., 2006; Deng et al., 1992; Duek and Fankhauser, 2003).

The inhibition of HY5 expression by COP1 (Oyama et al., 1997) was included in Eq. (18). Inactivation of CUL4 by COP1 (Eqs. (16) and (17)) was described by a Hill equation to account for the observed dimerization of COP1 protein complexes with SPA proteins and CUL4 (Chen et al., 2010; Yi and Deng, 2005; Zhu et al., 2008). The rest of the reactions were described by mass action kinetics for simplicity. The *θ*(*t*) function was used to achieve the smooth transitions between *L* and *D* in simulations of the diurnal conditions analogous to (Pokhilko et al., 2010)

L(t)=θ(t)=0.5⋅((1+tanh((t−24⋅floor(t/24)−dawn)/T))−(1+tanh((t−24⋅floor(t/24)−dusk)/T))+(1+tanh((t−24⋅floor(t/24)−24)/T)))

where *dawn* and *dusk* are the phases of dawn and dusk (normally *dawn=0*); *T* is the duration of twilight (we used *T*=0.05 h); tanh and floor—standard functions of hyperbolic tangent and rounding operation.

The equations were solved using MATLAB. To simulate the sharp changes in COP1 and CUL4 activities upon dark/light transitions (Figs. 4–6), we integrated the equations with the stiff solver ode15 s (The MathWorks UK, Cambridge). The parameters of the model were fitted to the timecourses of HFR1 and HY5 proteins, HY5 mRNA and COP1 protein abundance upon dark/light and light/dark transitions (Fig. 1 and Duek et al., 2004; Osterlund et al., 2000; von Arnim and Deng, 1994; von Arnim et al., 1997) as discussed in the description of Figs. 3–5. The resulting optimal parameter values, which closely match the data (Figs. 3–5) are presented in Table A2. The detailed scheme of the full model reactions in SBGN format is presented on Fig. 7.

System analysis demonstrated the existence of one steady state under constant light (Fig. 8A) and constant dark (Fig. 8B) conditions and a limit cycle (Fig. 8C) under diurnal conditions with the entrainment of the system by light.

### Sensitivity analysis of the model parameters

The response of the full model to parameters perturbations was analyzed by calculating the peak value of HFR1 and HY5 proteins, the value of the minimal COP1 activity together with its time and steady state level of the total COP1 after dark-to-light transition. The relative deviation of these 5 characteristics from their normal levels under 10% increase of each parameter is shown in Fig. 9. The parameters, which had strong influence on the timing and value of COP1 minimum, also affected the HFR1 peak value, while HY5 peak value partially correlated with the steady state COP1 level. Fig. 9 shows that 10% changes in parameters resulted in less than 10% changes of the system kinetics, demonstrating robustness of the model to parameter variations. The most sensitive parameters, which affect more than one of the system characteristics are *k*_0_, *k*_1_, *p*_3_, *h*_1_, *h*_9_.

The change in the whole kinetics of HFR1, HY5 proteins and COP1 activity upon parameter variation is further demonstrated in Fig. 10. We chose one of the most sensitive parameter—the rate constant of COP1 inactivation by light (*k*_0_) to show its effect on the system kinetics after dark-to-light transition. The decrease of *k*_0_ resulted in a slowing down of the fall in COP1 activity (Fig. 10C), which prevented HFR1 accumulation (Fig. 10A) and delayed accumulation of HY5 (Fig. 10B).

## References

[bib1] Ang L.H., Chattopadhyay S., Wei N., Oyama T., Okada K., Batschauer A., Deng X.W. (1998). Molecular interaction between COP1 and HY5 defines a regulatory switch for light control of Arabidopsis development. Mol. Cell.

[bib2] Bosu D.R., Kipreos E.T. (2008). Cullin-RING ubiquitin ligases: global regulation and activation cycles. Cell Div..

[bib3] Chamovitz D.A., Wei N., Osterlund M.T., von Arnim A.G., Staub J.M., Matsui M., Deng X.W. (1996). The COP9 complex, a novel multisubunit nuclear regulator involved in light control of a plant developmental switch. Cell.

[bib4] Chen H., Huang X., Gusmaroli G., Terzaghi W., Lau O.S., Yanagawa Y., Zhang Y., Li J., Lee J.H., Zhu D., Deng X.W. (2010). Arabidopsis CULLIN4-damaged DNA binding protein 1 interacts with CONSTITUTIVELY PHOTOMORPHOGENIC1-SUPPRESSOR OF PHYA complexes to regulate photomorphogenesis and flowering time. Plant Cell.

[bib5] Chen H., Shen Y., Tang X., Yu L., Wang J., Guo L., Zhang Y., Zhang H., Feng S., Strickland E., Zheng N., Deng X.W. (2006). Arabidopsis CULLIN4 forms an E3 ubiquitin ligase with RBX1 and the CDD complex in mediating light control of development. Plant Cell.

[bib6] Deng X.W., Matsui M., Wei N., Wagner D., Chu A.M., Feldmann K.A., Quail P.H. (1992). COP1, an Arabidopsis regulatory gene, encodes a protein with both a zinc-binding motif and a G beta homologous domain. Cell.

[bib7] Duek P.D., Fankhauser C. (2003). HFR1, a putative bHLH transcription factor, mediates both phytochrome A and cryptochrome signalling. Plant J..

[bib8] Duek P.D., Elmer M.V., van Oosten V.R., Fankhauser C. (2004). The degradation of HFR1, a putative bHLH class transcription factor involved in light signaling, is regulated by phosphorylation and requires COP1. Curr. Biol..

[bib9] Fankhauser C., Chory J. (2000). RSF1, an Arabidopsis locus implicated in phytochrome A signaling. Plant Physiol..

[bib10] Fittinghoff K., Laubinger S., Nixdorf M., Fackendahl P., Baumgardt R.L., Batschauer A., Hoecker U. (2006). Functional and expression analysis of Arabidopsis SPA genes during seedling photomorphogenesis and adult growth. Plant J..

[bib11] Hong S.H., Kim H.J., Ryu J.S., Choi H., Jeong S., Shin J., Choi G., Nam H.G. (2008). CRY1 inhibits COP1-mediated degradation of BIT1, a MYB transcription factor, to activate blue light-dependent gene expression in Arabidopsis. Plant J..

[bib12] Jang I.C., Yang S.W., Yang J.Y., Chua N.H. (2007). Independent and interdependent functions of LAF1 and HFR1 in phytochrome A signaling. Genes Dev..

[bib13] Jang S., Marchal V., Panigrahi K.C., Wenkel S., Soppe W., Deng X.W., Valverde F., Coupland G. (2008). Arabidopsis COP1 shapes the temporal pattern of CO accumulation conferring a photoperiodic flowering response. Embo. J..

[bib14] Jenkins G.I. (2009). Signal transduction in responses to UV-B radiation. Annu. Rev. Plant Biol..

[bib15] Jiao Y., Lau O.S., Deng X.W. (2007). Light-regulated transcriptional networks in higher plants. Nat. Rev. Genet..

[bib16] Jiao Y., Ma L., Strickland E., Deng X.W. (2005). Conservation and divergence of light-regulated genome expression patterns during seedling development in rice and Arabidopsis. Plant Cell.

[bib17] Khanna R., Shen Y., Toledo-Ortiz G., Kikis E.A., Johannesson H., Hwang Y.S., Quail P.H. (2006). Functional profiling reveals that only a small number of phytochrome-regulated early-response genes in Arabidopsis are necessary for optimal deetiolation. Plant Cell.

[bib18] Khanna R., Shen Y., Marion C.M., Tsuchisaka A., Theologis A., Schafer E., Quail P.H. (2007). The basic helix-loop-helix transcription factor PIF5 acts on ethylene biosynthesis and phytochrome signaling by distinct mechanisms. Plant Cell.

[bib19] Kiba T., Henriques R., Sakakibara H., Chua N.H. (2007). Targeted degradation of PSEUDO-RESPONSE REGULATOR5 by an SCFZTL complex regulates clock function and photomorphogenesis in Arabidopsis thaliana. Plant Cell.

[bib20] Kim W.Y., Fujiwara S., Suh S.S., Kim J., Kim Y., Han L., David K., Putterill J., Nam H.G., Somers D.E. (2007). ZEITLUPE is a circadian photoreceptor stabilized by GIGANTEA in blue light. Nature.

[bib21] Li Q.H., Yang H.Q. (2007). Cryptochrome signaling in plants. Photochem. Photobiol..

[bib22] Locke J.C., Kozma-Bognar L., Gould P.D., Feher B., Kevei E., Nagy F., Turner M.S., Hall A., Millar A.J. (2006). Experimental validation of a predicted feedback loop in the multi-oscillator clock of *Arabidopsis thaliana*. Mol. Syst. Biol..

[bib23] Lyapina S., Cope G., Shevchenko A., Serino G., Tsuge T., Zhou C., Wolf D.A., Wei N., Shevchenko A., Deshaies R.J. (2001). Promotion of NEDD-CUL1 conjugate cleavage by COP9 signalosome. Science.

[bib24] Ma L., Gao Y., Qu L., Chen Z., Li J., Zhao H., Deng X.W. (2002). Genomic evidence for COP1 as a repressor of light-regulated gene expression and development in Arabidopsis. Plant Cell.

[bib25] Monte E., Al-Sady B., Leivar P., Quail P.H. (2007). Out of the dark: how the PIFs are unmasking a dual temporal mechanism of phytochrome signalling. J. Exp. Bot..

[bib26] Osterlund M.T., Hardtke C.S., Wei N., Deng X.W. (2000). Targeted destabilization of HY5 during light-regulated development of Arabidopsis. Nature.

[bib27] Oyama T., Shimura Y., Okada K. (1997). The Arabidopsis HY5 gene encodes a bZIP protein that regulates stimulus-induced development of root and hypocotyl. Genes Dev..

[bib28] Pokhilko A., Hodge S.K., Stratford K., Knox K., Edwards K.D., Thomson A.W., Mizuno T., Millar A.J. (2010). Data assimilation constrains new connections and components in a complex, eukaryotic circadian clock model. Mol. Syst. Biol..

[bib29] Saijo Y., Sullivan J.A., Wang H., Yang J., Shen Y., Rubio V., Ma L., Hoecker U., Deng X.W. (2003). The COP1-SPA1 interaction defines a critical step in phytochrome A-mediated regulation of HY5 activity. Genes Dev..

[bib30] Schwechheimer C., Serino G., Callis J., Crosby W.L., Lyapina S., Deshaies R.J., Gray W.M., Estelle M., Deng X.W. (2001). Interactions of the COP9 signalosome with the E3 ubiquitin ligase SCFTIRI in mediating auxin response. Science.

[bib31] Seo H.S., Yang J.Y., Ishikawa M., Bolle C., Ballesteros M.L., Chua N.H. (2003). LAF1 ubiquitination by COP1 controls photomorphogenesis and is stimulated by SPA1. Nature.

[bib32] Suzuki G., Yanagawa Y., Kwok S.F., Matsui M., Deng X.W. (2002). Arabidopsis COP10 is a ubiquitin-conjugating enzyme variant that acts together with COP1 and the COP9 signalosome in repressing photomorphogenesis. Genes Dev..

[bib33] Van Cauter E., Hardman J.G., Dumont J.E. (1976). Implications of cross inhibitory interactions of potential mediators of hormone and neurotransmitter action. Proc. Natl. Acad. Sci. USA.

[bib34] Vandenbussche F., Habricot Y., Condiff A.S., Maldiney R., Van der Straeten D., Ahmad M. (2007). HY5 is a point of convergence between cryptochrome and cytokinin signalling pathways in *Arabidopsis thaliana*. Plant J..

[bib35] von Arnim A.G., Deng X.W. (1994). Light inactivation of Arabidopsis photomorphogenic repressor COP1 involves a cell-specific regulation of its nucleocytoplasmic partitioning. Cell.

[bib36] von Arnim A.G., Osterlund M.T., Kwok S.F., Deng X.W. (1997). Genetic and developmental control of nuclear accumulation of COP1, a repressor of photomorphogenesis in Arabidopsis. Plant Physiol..

[bib37] Wang H., Ma L.G., Li J.M., Zhao H.Y., Deng X.W. (2001). Direct interaction of Arabidopsis cryptochromes with COP1 in light control development. Science.

[bib38] Wei N., Serino G., Deng X.W. (2008). The COP9 signalosome: more than a protease. Trends Biochem. Sci..

[bib39] Wei N., Kwok S.F., von Arnim A.G., Lee A., McNellis T.W., Piekos B., Deng X.W. (1994). Arabidopsis COP8, COP10, and COP11 genes are involved in repression of photomorphogenic development in darkness. Plant Cell.

[bib40] Yanagawa Y., Sullivan J.A., Komatsu S., Gusmaroli G., Suzuki G., Yin J., Ishibashi T., Saijo Y., Rubio V., Kimura S., Wang J., Deng X.W. (2004). Arabidopsis COP10 forms a complex with DDB1 and DET1 in vivo and enhances the activity of ubiquitin conjugating enzymes. Genes Dev..

[bib41] Yang H.Q., Tang R.H., Cashmore A.R. (2001). The signaling mechanism of Arabidopsis CRY1 involves direct interaction with COP1. Plant Cell.

[bib42] Yang J., Lin R., Hoecker U., Liu B., Xu L., Wang H. (2005). Repression of light signaling by Arabidopsis SPA1 involves post-translational regulation of HFR1 protein accumulation. Plant J..

[bib43] Yi C., Deng X.W. (2005). COP1—from plant photomorphogenesis to mammalian tumorigenesis. Trends Cell Biol..

[bib44] Yu J.W., Rubio V., Lee N.Y., Bai S., Lee S.Y., Kim S.S., Liu L., Zhang Y., Irigoyen M.L., Sullivan J.A., Zhang Y., Lee I., Xie Q., Paek N.C., Deng X.W. (2008). COP1 and ELF3 control circadian function and photoperiodic flowering by regulating GI stability. Mol. Cell.

[bib45] Zhang X.N., Wu Y., Tobias J.W., Brunk B.P., Deitzer G.F., Liu D. (2008). HFR1 is crucial for transcriptome regulation in the cryptochrome 1-mediated early response to blue light in *Arabidopsis thaliana*. PLoS One.

[bib46] Zhu D., Maier A., Lee J.H., Laubinger S., Saijo Y., Wang H., Qu L.J., Hoecker U., Deng X.W. (2008). Biochemical characterization of Arabidopsis complexes containing constitutively photomorphogenic1 and suppressor of phya proteins in light control of plant development. Plant Cell.

